# Association of Health and Food Expenditures Inequality With Health Outcomes: A Case Study on Iranian Rural Households

**DOI:** 10.5812/ircmj.14335

**Published:** 2014-03-05

**Authors:** Seyran Naghdi, Hesam Ghiasvand, Nasrin Shaarbafchi Zadeh, Saeidreza Azami, Tayebeh Moradi

**Affiliations:** 1 Research Center for Health Services Management, Institute for Futures Studies in Health, Kerman University of Medical Sciences, Kerman, IR Iran; 2 Hospital Management Research Center, Iran University of Medical Sciences, Tehran, IR Iran; 3 Health Management and Economics Research Center, School of Health Management and Information Sciences, Iran University of Medical Sciences, Tehran, IR Iran

**Keywords:** Health Expenditures, Socioeconomic Factors, Health status

## Abstract

**Background:**

Inequality in households’ and individuals' consumption expenditures is one of the most important aspects of health status difference among households and individuals.

**Objectives:**

We investigated the impact of some macro-economic factors specially inequality factors on the Iranian rural health status since 1986 through 2012.

**Patients and Methods:**

We conducted a longitudinal ecological and analytical study. The average sample size was 14602 households whom Iranian Statistics Center selected by a multi-stages clustering sampling approach. All required data has been collected from Iranian Statistics Centre and Deputy for Curial Affaires of Iranian Ministry of Health. We calculated the Gini coefficients for the rural food and health expenditures, then conducted a transloge autoregressive order one (AR1) to investigate the association between the Iranian rural households' key mortality rates and the food and health expenditure Gini coefficients, time trend, GDP per capita (PPP), and GDP per capita Gini coefficients.

**Results:**

The mean of Gini coefficients were 0.137 and 0.21 for the rural food expenditures inequality based on current and constant price, respectively. In addition, the mean of Gini coefficients were 0.26 and 0.31 for the rural health expenditures inequality based on current and constant price, respectively. The time trend, transloged form of Gini coefficients for health expenditures and GDP per capita Gini coefficients presented a significant negative correlation with transloged form of neonatal mortality rate. With regard to the transloged form of under five mortality we observed a significant negative correlation with time trend and transloged form of Gini coefficients for health expenditure and GDP per capita. Finally, there was a significant negative correlation between transloged forms of maternal mortality rate.

**Conclusions:**

Iranian policy makers should consider the rural health and food expenditures inequality and try to adopt more effective policies and plans to decrease it. In addition, they should improve the macro-economic factors to improve the rural households' health status.

## 1. Background

One of the most important controversial issues for health policy makers is equity in health and equal access to health services among different socio-economic groups (1). Since the publication of Black's report in England in 1980, many studies have been conducted that concerned the influence of social-economic factors on health status of the population (2). Considerable inequalities in the health sector are known as one of the major concerns in both developed and developing countries (3). No country in the world is immune to health inequality, but the main concern relates to the growing trend of inequalities among different socio-economic groups (4). Differences in health status of social groups are related to inequalities in working conditions, lifestyles, and socio-economic policies of each country. Even in the richest countries, the poor have lower health level, lower life expectancy, and higher morbidity rates than the rich (5). Studies have shown that the mortality rate difference in different social classes is not only because of individual differences, but also due to inequality in the structure that people live in it, which has an important role in this context (6). A study from 1998 to 2007 reported significant inequality in healthcare expenditures for households of Tehran and with a range from 0.6 to 0.8 based on Gini coefficient (7).

Moreover, other studies found an association between income and socio-economic status with health status and concluded that income inequality had a significant impact on population, households, and individuals health circumstance (8-13). In most studies, the most important issue concerning the association between economic status of households and their health level was to consider an appropriate representative variable with desirable features in order to show economic household status. Recently, the researchers suggested the households’ consumption expenditure as the representative for households' economic status variable. This is due to two reasons; first, the households’ consumption expenditures can be an indicative variable for households’ capacity to pay. Second, the estimation of expenditures is easier than income levels, because people often do not declare their real income in developing and poor countries (13).

Inequality as a basic principle for welfare services including education, health, housing, and other life necessities, had always a special position in Iranian national programs and policies. Iranian Constitutional law and 20-year vision document state: “the government must provide basic health services for all people”. In four articles of Constitutional law of the Islamic Republic of Iran (3, 15, 29, and 100) state: "the government must provide health, education, and welfare services for all people of the country without any discrimination" (14).

## 2. Objectives

This study investigated the distribution of food and health expenditures among the Iranian rural households, and its association with health status of these households during 1986 to 2012.

## 3. Patients and Methods

This longitudinal ecological study was conducted in a retrospective design. Study population included all the Iranian rural households separated by province during 1986 to 2012. Sampling of this research was based on method and sample size of Iranian Statistics Center. This center utilizes a three stages clustering sampling method to collect data. Annually Iranian Statistics Center conducts surveys to investigate income and cost status of the urban and rural households in the national Scale. Then, a questionnaire was designed and completed by direct interviewing households’ head. The sample of the survey was carried out in a three-stage sampling process. In the first stage the geographic regions was chosen, in the second stage the clusters and in the third stage the households were selected (15). The sample size was not constant during the study period and it was variable in each year. On average, sample size of the rural households was 14602 households during 1986 to 2012.

### 3.1. Tools and Methods of Data Collection

All data were collected using a self-administered form including the name of variables separated by the rural areas of all Iranian provinces. In addition, the rural health indicators were collected from the Iranian Ministry of Health deputy for Health Affairs and Vital Horoscope of the rural population was received. All data collected through an internet search and did not need any special ethical certification or approval to use. 

### 3.2. Study Variables

We used the infant mortality rate, under-five children's mortality and maternal mortality rates, rural health and food expenditures Gini coefficients, GDP per capita adjusted for purchasing power parity, Gini coefficient for GDP per capita, and time trend in this study. The mortality indicators were selected based on their popularity and importance in assessing the health systems' performance in addition to the availability of the regular and reliable concerning.

The rural households' average expenditures of food was defined as the monetary value of payments for food by the rural households during a particular year. According to Iranian Statistics Center, these costs encompassed flour, crops, bread and biscuits, meat, milk, milk products, eggs, oils and fats, fresh fruits, fresh vegetables, dried fruits and nuts, bean, canned food, vegetable products, sugar, confectionery, jam, tea, coffee, cocoa, all kinds of spices, sauces and other food ingredients, and beverage (15). The rural households average expenditure of health was defined as the monetary value of payments for health care and health insurance by the rural households during a year (15). Other definitions are as following:

Maternal mortality rate: The number of maternal deaths due to pregnancy or pregnancy complications per 100,000 live births (16).

Infant mortality rate: The number of deaths of children under one month old per 1,000 live births (16).

Under-five children's mortality rate: The number of under-five-year old mortality per 1,000 live births (16).

GDP per capita adjusted for purchasing power parity: This means the converted GDP per capita to international dollars using purchasing power parity rates. An international dollar has the same purchasing power over GDP as US dollar has in the United States (17).

### 3.3. Data Analysis

**Figure 1.**
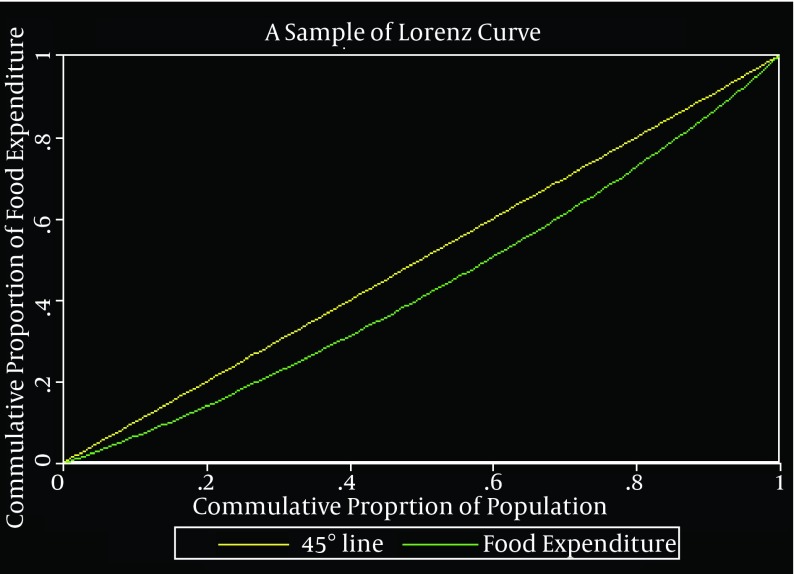
A Simple Lorenz Curve Schematic Form

We conducted a four-phase process to analyze the data. In the first phase, we calculated the Gini coefficient to determine the rural households’ food and health expenditures distribution. We considered two scenarios in this stage; 1) Calculating Gini coefficient based on current prices, and 2) Calculating Gini coefficient based on constant prices. The reason of considering above scenarios was investigation of inflation effects on Gini coefficients levels. Gini coefficient is calculated based on Lorenz curve and classified in relative inequality indices group. Lorenz curve was composed of two orthogonal axes, which is located on the horizontal axis of cumulative frequency of population and the vertical axis of cumulative frequency (cumulative proportion) of the variable for distribution (e.g. income or expenditure in our discussion). The two axes are divided into two equal 45-degree parts using a bisector, which is called perfect equality line, because all the points on it represent identical values on vertical and horizontal axes. Now if we draw the actual and current distribution status of a particular variable on a curve, it can be determined that how much deviation from the ideal or desired situation exists for distribution of that variable among population groups. When the gap between the perfect equality line and the Lorenz curve increases, it indicates that there is a greater inequality in the distribution of this variable. The Gini coefficient is also equal to the area between the perfect equality line and the Lorenz curve, which ranges from zero to one. Zero represents perfect equality in the distribution of interest variable and one shows perfect inequality in the distribution of interest variable. Figure 1, presents a simple Lorenz curve and extraction method of the Gini coefficient are indicated (13).

In the second phase, in order to present the food and health importance among the Iranian rural households, we calculated the share of expenditures based on constant price, which households spent for these items in their consumption expenditures. For this purpose the formula (3) was used Equation 1:

$$\mathrm{Rual}\mathrm{Houshol}s^{'}\mathrm{Food}(\mathrm{Health})\mathrm{Expenditure}\mathrm{Share}=\frac{\mathrm{The}\mathrm{Quantity}\mathrm{of}\mathrm{Payment}\mathrm{For}\mathrm{Food}(\mathrm{Health})\mathrm{During}\mathrm{One}\mathrm{Year}}{\mathrm{The}\mathrm{Total}\mathrm{Payments}\mathrm{For}\mathrm{All}\mathrm{Goods}\mathrm{and}\mathrm{Services}\mathrm{during}\mathrm{One}\mathrm{Year}}$$

This share is calculated for the period of 1986 to 2012 and based on constant prices. In third phase, we compared the calculated Gini coefficient in two scenarios of current price and constant price using paired samples t test. The aim was to investigate the inflation association with inequality of food and health expenditure among the rural households. In order to convert the current variables, such as the rural food and health expenditures, to the constant expenditures, current values of these variables in each year should divide by on Price index in that year. Iranian Statistics Center adapted the 2002 as the base year for the rural households; by referring to that year, we found that the price index was 100 in 2002.

Finally, we examined the impact of food and health expenditures inequality alongside the time trend, Iranian GDP per capita based on purchasing power parity (PPP), and Gini coefficients for GDP per capita on the rural households’ health status. We conducted a translog first order autoregressive (AR) model (1). We employed this regression form because there were several classic assumptions problems in our data. The linearity, multicollinearity, heteroskedasticity, normality, and serial auto-correlation were tested and the suitable fixing methods was executed. The aforementioned variables were considered because the health status is influenced by many factors including social and economic factors and regarding to limitation for accessibility to social factors, we used some economic factors. The Gini coefficient, statistical tests, and regression were estimated by STATA software version 11.

## 4. Results

**Figure 2.**
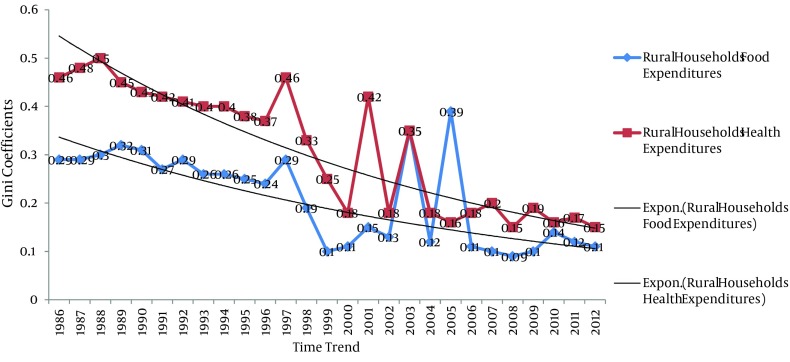
Gini Coefficients for the Rural Households’ Food and Health Expenditures (Based Constant Price)

In order to describe the food and health status, Gini coefficients for food and health costs were calculated and separated by two scenarios of current and constant prices. The results are presented in Table 1. In addition, the Figure 2. Presents the trend of Gini coefficients for the rural food and health expenditures over the considered time.

**Table 1.** Gini Coefficients for Rural Households’ Food and Health Expenditures

Years	Rural Households' Food Expenditures		Rural Households' Health Expenditures	
	Current Price	Constant Price	Current Price	Constant Price
**1986**	0.2	0.29	0.36	0.46
**1987**	0.18	0.29	0.35	0.48
**1988**	0.17	0.3	0.35	0.5
**1989**	0.16	0.32	0.34	0.45
**1990**	0.15	0.31	0.33	0.43
**1991**	0.16	0.27	0.32	0.42
**1992**	0.15	0.29	0.32	0.41
**1993**	0.16	0.26	0.33	0.4
**1994**	0.14	0.26	0.32	0.4
**1995**	0.14	0.25	0.31	0.38
**1996**	0.13	0.24	0.3	0.37
**1997**	0.12	0.29	0.28	0.46
**1998**	0.13	0.19	0.28	0.33
**1999**	0.1	0.1	0.23	0.25
**2000**	0.11	0.11	0.17	0.18
**2001**	0.12	0.15	0.18	0.42
**2002**	0.13	0.13	0.18	0.18
**2003**	0.12	0.35	0.35	0.35
**2004**	0.15	0.12	0.19	0.18
**2005**	0.1	0.39	0.16	0.16
**2006**	0.11	0.11	0.18	0.18
**2007**	0.1	0.1	0.2	0.2
**2008**	0.12	0.09	0.2	0.15
**2009**	0.13	0.1	0.22	0.19
**2010**	0.13	0.14	0.21	0.16
**2011**	0.14	0.12	0.22	0.17
**2012**	0.15	0.11	0.23	0.15
**Mean**	0.137	0.21	0.26	0.31

**Figure 3.**
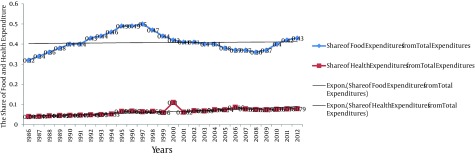
The Proportion of Food and Health Expenditure as a Share of Iranian Rural Households’ Consumption Expenditures

Figure 2, presents a decreasing trend for the Iranian rural households food and health expenditures over the time. Furthermore, we presented the share of the rural households' food and health expenditure based on constant price from the rural households' consumption expenditure. The results of the calculations are presented in Table 2 and Figure 3 as below. The time trend for food and health expenditure share is illustrated in Figure 3. 

**Table 2.** The Iranian Rural Households' Food and Health Expenditures Ratios 1986-2012

Year	Total Consumption Expenditures	Total Food Expenditures	Total Health Expenditures	Share of Food Expenditures from Total Expenditures	Share of Health Expenditures from Total Expenditures
**1986**	6793127	2173210	269218	0.32	0.04
**1987**	6977334	2391721	288420	0.34	0.041
**1988**	7219720	2603483	318319	0.36	0.044
**1989**	77552073	2896531	341563	0.38	0.045
**1990**	7871982	3198934	360606	0.4	0.046
**1991**	8240372	3296522	385402	0.4	0.047
**1992**	8403956	3592191	414092	0.43	0.049
**1993**	8709291	3820251	437183	0.44	0.05
**1994**	8652950	4008521	461091	0.46	0.053
**1995**	9002523	4402312	595231	0.49	0.066
**1996**	9412085	4602201	630014	0.49	0.067
**1997**	10085301	5012619	651025	0.5	0.064
**1998**	10803836	5131586	716811	0.47	0.066
**1999**	13637271	6087403	828485	0.44	0.06
**2000**	15673261	6633246	1757091	0.42	0.11
**2001**	17232892	7070294	1073043	0.41	0.062
**2002**	21394955	8780087	1492122	0.41	0.069
**2003**	25676034	10227852	1757091	0.4	0.068
**2004**	33543771	13030920	2503765	0.4	0.074
**2005**	37502952	14313100	2787975	0.38	0.074
**2006**	41569925	15512672	3583501	0.37	0.086
**2007**	48846045	18202733	3849659	0.37	0.078
**2008**	52809649	19140763	3970381	0.36	0.075
**2009**	55914031	21039862	4150822	0.37	0.074
**2010**	58612906	23709215	4470237	0.4	0.076
**2011**	59107389	25023891	4619361	0.42	0.078
**2012**	61290432	26193401	4866210	0.43	0.079
**Mean**	24168002	9707241	1762174	0.41	0.065

In an overall view, we observed a relative constant trend about the Iranian rural food and health expenditures ratios during 1986 to 2012. Was there any significant different between the Iranian rural households' food and health expenditures based on current prices in comparison with constant prices? We answered this question through conducting a paired t-test, which is presented in Table 3. 

**Table 3.** The Comparison Between the Iranian Rural Food and Health Expenditures Inequality Based on Current Prices and Constant Prices

	Mean ± SD	Mean ± SE	CI (95%)	t-test	Significant
**Difference between rural households’ food expenditures on current price with constant price**	-0.046 ± 0.104	-0.046 ± 0.03	0.02-0.112	-1.521	0.16
**Difference between rural households’ health expenditures on current price with constant price**	-0.02 ± 0.074	-0.046 ± 0.02	0.028-0.066	-0.9	0.38

Finally, we investigated the economic determinants of the Iranian rural households' mortality rates. We presented the regression models separately with their diagnostic tests for classic assumptions and fixed any probable problems through available fixing methods. The first regression was related to the neonatal mortality rate determinants factors. In first step, we observed a dispersed pattern in scatter plot for neonatal mortality rate against four explanatory variables except the time trend, which presented a straightforward positive dispersion pattern. Therefore, we transformed all variables (except time trend) to natural logarithm form. Then we rerun the scatter plot for these variables and observed a relatively linear dispersion. Second, we investigated the multicollinearity between explanatory variables and the VIF indices (tolerance quantities) were less than ten (or more than 0.1); hence, there was not any multicollonearity between considered variables. Third, the normality of residuals was test by Skewness-Kurtosis test; the results of the probability of skewness was 0.544 with significance level about 0.44 (P = 0.44) and the probability of Kurtosis was 0.8 with significance level about 0.8 (P = 0.8). Therefore, we did not observe any non-normality in the regression. Forth, the heteroskedasticity was assessed and the Cook-Weisberg statistics based on χ2 test was 0.8 with the significance level of 0.37 (P = 0.37). Fifth, the autocorrelation between residuals was tested. The Durbin-Watson statistic was about 0.85 that implies a positive successive correlation between residuals. Hence, to fix this problem, we conducted the Paris-Winsten first order autoregressive (AR-1). Table 4 presents the neonatal mortality regression results:

**Table 4.** Paris-Winsten AR (1) Iterated Estimation of Neonatal Mortality Rate Regression ^a^

Ln NMR	Coefficient	SE	t-statistics	P Value > |t|	CI
**Time trend**	-0.026	0.004	-5.55	00	-0.036-0.017
**Ln GiniFood**	-0.011	0.03	-0.36	0.72	-.05-0.074
**Ln GiniHealth**	-0.04	0.043	-3.05	0.035	-.131-0.05
**LnGDP (PPP)**	-.013	0.21	-0.65	0.52	-0.057-0.03
**Ln GDPGINI**	-0.23	0.2	2.17	0.045	-0.18-0.65
**Constant**	14.95	2.37	6.43	00	3.65-31.23

^a^ Rho = 0.642; Durbin-Watson statistic (original) = 0.85; Durbin-Watson statistic (transformed) = 1.77; Adjusted R^2^ = 0.97 F (5, 21) = 184.5; Prob > F = 0.0

Second regression was conducted to investigate the determinants of under-five children’s mortality rate. The diagnostic tests were performed for every probable classic assumptions problems. The linearity test reflected a dispersed pattern except for time trend in which a negative linear pattern for time trend was seen. Therefore, we transformed the dependent and explanatory variables to a logarithmic form with the exception of the time trend. We repeated the linearity test and did not observe a high-dispersed pattern. The VIF index results showed there was not any multicollinearity between explanatory variables (VIF < 10 or tolerance > 0.1). The Skewness-Kurtosis test presented a normal distribution for residuals quantities (the Pr skewness was 0.61 and the Pr Kurtosis was 0.071). In addition, the Cook-Weisberg χ2 test was 0.02 with significant level of about 0.88 that meant there was not any heteroskedasticity for residual quantities. However, the Durbin-Watson statistic was about 1.14 and this implied that there was a positive serial autocorrelation between residuals. Hence, we conducted the Paris-Winsten AR (1) for fixing this problem. The regression results for under five children’s mortality rate are presented in Table 5. 

**Table 5.** Paris-Winsten AR (1) Iterated Estimation of Under Five Mortality Rate Regression ^a^

LnUFMR	Coefficient	SE	t-statistics	P > |t|	CI
**Time trend**	-0.043	0.005	-8.2	00	-0.053-0.032
**Ln GiniFood**	-0.006	0.037	-0.02	1	-0.077-0.76
**Ln GiniHealth**	-0.062	0.053	-3.18	0.025	-0.17-0.05
**LnGDP (PPP)**	-0.002	0.025	-0.08	0.93	-0.055-0.051
**Ln GDPGINI**	-0.045	0.24	-4.19	0.08	-0.002-0.04
**Constant**	12.75	6.31	9.65	00	2.65 20.43

^a^ Rho = 0.61; Durbin-Watson statistic (original) = 1.14; Durbin-Watson statistic (transformed) = 1.9; Adjusted R^2^ = 0.97; F (5, 21) = 183.46; Prob > F = 0.0

The last regression analysis was conducted for the determinants factors of maternal mortality rate. In this regression such as previous regressions, we firstly tested the classic assumptions. The scatter plot presented a relative order for time trend, but this was not true for other explanatory variables and therefore, we transformed them into a logarithmic regression. The VIF indices (tolerance quantities) were below ten (higher than 0.1) and this meant there was not any multicollinearity between explanatory variables. The Skewness-Kurtosis statistics probabilities were 0.12 and 0.81, respectively and this indicated that there was a normal distribution of the residuals. The Cook-Weisberg statistics based on χ2 was 1.12 with probability of about 0.23 (P = 0.23); therefore, we did not observe heteroskedasticity for residual. However, the Durbin-Watson statistic was equal to 1.1 that indicated a positive autocorrelation between residuals. Hence, we conducted a Paris-Winsten AR (1) regression. The results of regression are presented in Table 6. 

**Table 6.** Paris-Winsten AR Iterated Estimation of Maternal Mortality Rate Regression ^a^

LnMMR	Coefficients	SE	t-statistics	P > |t|	CI
**Year**	-0.24	0.305	-6.6	00	-2.63-1.37
**Ln GiniFood**	-0.003	0.031	-2.03	0.12	-0.00042-0.04
**Ln GiniHealth**	-0.04	0.05	-3.45	0.031	-0.002-0.35
**LnGDP (PPP)**	-0.003	0.55	-5.15	0.074	-0.0006-0.25
**Ln GDPGINI**	-0.0005	0.0065	-0.54	1	-0.00003-0.061
**Constant**	43.25	11.74	15.74	00	2.35-85.54

^a^ Rho = 0.6; Durbin-Watson statistic (original) = 1.1; Durbin-Watson statistic (transformed) = 1.85; Adjusted R^2^ = 0.87; F (5, 21) = 371.5; Prob > F = 0.0

## 5. Discussion

The rural households’ health and food expenditure inequality was not very substantial and significant; this implied a light difference amongst different rural areas in country. However, the calculated annual values for the rural households’ food and health expenditure in some years indicate major differences. Although when inequality considered based on constant price, these differences have an obvious and concerning pattern. The mean of Gini coefficient based on current price for food expenditure was about 0.137. On the other hand, the calculated mean value for Gini coefficient based on constant price was about 0.21. In addition, the means of Gini coefficient were about 0.26 and 0.31 based on current price and constant price for the rural households' health expenditures, respectively. The results of a study indicated that inequality of health expenditure for the Iranian rural households based on Gini coefficient from 1995 to 2005 was about 0.4 in average and based on concentration index it was about 0.5. These indices have been calculated for urban areas about 0.38 and 0.5 based on Gini coefficient and concentration index, respectively (18). moreover, another study on the rural and urban households of Tehran concluded that the Gini coefficients were 0.6 to 0.8 for health expenditure that indicated a high level of inequality during 1989 to 2007; however, this was lower for food expenditure as Gini coefficient values were between 0.3 to 0.4 (7). In first one of the above studies, the researchers considered the health and food expenditures inequality between income deciles and its geographical aspect was not considered as a purpose of the study. In addition, the second study only considered the rural and urban areas of Tehran; hence, the inequality levels in both studies were different with our study. However, in limitation section of the second study the authors stated that there had been many missing data during the study and therefore, probably it was one of the reasons for the high level of inequality in that study. Furthermore, in Iranian Statistics survey on households, all households’ data on health expenditures even cosmetic surgery, services, and caesarian delivery were collected. This expenditure is not considered vital and promotional for households’ health status and might indicate a higher level of expenditure inequality. The results of a study conducted in Malaysia showed that the health expenditure inequality in that country was mild and slight and the richer households paid more expenditure and imposed heavier burden of financial of health services (19).

Based on the results shown in Table 2, food expenditure constructed a considerable share (0.41) of the rural households' consumption expenditure; in contrast, the health expenditures was less contributed in consumption expenditure (0.065) in the rural households. This might reflect that the food was accounted as an essential commodity for survival of households and on the contrary, healthcare was a secondary and complementary need for households. In other words, food constructed a major part of annual budget of the rural households. As a result, it is possible that health as an important welfare commodity, especially for more vulnerable groups, remained ignored and unsatisfied. According to the results of paired t-test, the food and health expenditures inequality based on current price with constant price showed no significant differences during the studied period (P = 0.16). In fact, the food and health inequality levels were dependent to the inflation rate and in both current and constant scenarios, this inequality remains alight with the exception of some years. The three regressions presented relative similar results, for each three models the time trend has a negative statistical relationship with mortality rates (P = 000). In addition, the Gini for health expenditures has a negative statistical relationship with mortality rate (P < 0.05). The Gini for food expenditure and GDP per capita did not present a statistically significant association with the mortality rates (P > 0.05). In addition, the Gini coefficients for GDP per capita presented a statistically significant association with the neonatal and under-five children’s mortality rates (P < 0.05). Moreover, with regard to time series data and conducting an autoregressive model to determine the determinant factors of the rural households' mortality rates, the previous levels of independent variables influenced the current mortality rates. It appeared that inequality in food expenditure had less impact on the health status in comparison to the inequality in health expenditure. However, it should be noted that in addition to playing an important role in survival, food is effective on overall health of the society as well; Thus, its role on health of the society, especially vulnerable groups, should not be neglected just because of focusing on figures of statistical analysis. The results of a study conducted by Hong et al. in Bangladesh showed that inequality in wealth of households had a strong significant association with inadequate growth of children. In other words, in contrast to the richest quintile of this country, the children in the poorest quintile in Bangladesh suffered three times more from complications related to poor growth (OR = 3.6%: CI = 3-4.3) (12). in addition, in another study by Karim et al. a significant association was seen amongst different groups of poor people with benefit levels and subsequently, the health status (4). Inequality, especially in fields related to well-being of a society such as health or food, causes adverse effect on population health status. Inequality in distribution of health and food costs status in the rural areas of our country are not very substantial; it should be noted that a further reduction of this amount will have a positive effect on society and can be considered as a great achievement to human development in our country. Another conclusion of this study is that regarding the small contribution of the rural households' health expenditures in total consumption expenditure, as well as high association between distributions of these costs with health consequences in this population, more inequality in mentioned expenditure would have no outcomes but further deterioration in health status of the rural population. This is the key point that should be of special interest to policy makers and planners of country. Our study had some limitation in execution; regarding the period of this study, the information about South, Razavi, and North Khorasan provinces were considered as one province, namely, Khorasan. On the other hand, data associated with some provinces in some years were not available; hence, the researchers estimated the probable data according to the time trend of variables.
